# Dynamic and Static Overloading Induce Early Degenerative Processes in Caprine Lumbar Intervertebral Discs

**DOI:** 10.1371/journal.pone.0062411

**Published:** 2013-04-30

**Authors:** Cornelis P. L. Paul, Tom Schoorl, Hendrik A. Zuiderbaan, Behrouz Zandieh Doulabi, Albert J. van der Veen, Peter M. van de Ven, Theo H. Smit, Barend J. van Royen, Marco N. Helder, Margriet G. Mullender

**Affiliations:** 1 Department of Orthopaedic Surgery, VU University Medical Center, Amsterdam, The Netherlands; 2 Research Institute MOVE, Faculty of Human Movement Sciences, VU University Amsterdam, Amsterdam, The Netherlands; 3 Skeletal Tissue Engineering Group Amsterdam, VU University Medical Center, Amsterdam, The Netherlands; 4 Department of Oral Cell Biology, Academic Centre of Dentistry Amsterdam, Amsterdam, The Netherlands; 5 Department of Epidemiology and Biostatistics, VU University Medical Center, Amsterdam, The Netherlands; 6 Department of Plastic, Reconstructive and Hand Surgery, VU University Medical Center, Amsterdam, The Netherlands; National Centre for Scientific Research, 'Demokritos', Greece

## Abstract

Mechanical overloading of the spine is associated with low back pain and intervertebral disc (IVD) degeneration. How excessive loading elicits degenerative changes in the IVD is poorly understood. Comprehensive knowledge of the interaction between mechanical loading, cell responses and changes in the extracellular matrix of the disc is needed in order to successfully intervene in this process. The purpose of the current study was to investigate whether dynamic and static overloading affect caprine lumbar discs differently and what mechanisms lead to mechanically induced IVD degeneration. Lumbar caprine IVDs (n = 175) were cultured 7, 14 and 21 days under simulated-physiological loading (control), high dynamic or high static loading. Axial deformation and stiffness were continuously measured. Cell viability, cell density, and gene expression were assessed in the nucleus, inner- and outer annulus. The extracellular matrix (ECM) was analyzed for water, glycosaminoglycan and collagen content. IVD height loss and changes in axial deformation were gradual with dynamic and acute with static overloading. Dynamic overloading caused cell death in all IVD regions, whereas static overloading mostly affected the outer annulus. IVDs expression of catabolic and inflammation-related genes was up-regulated directly, whereas loss of water and glycosaminoglycan were significant only after 21 days. Static and dynamic overloading both induced pathological changes to caprine lumbar IVDs within 21 days. The mechanism by which they inflict biomechanical, cellular, and extracellular changes to the nucleus and annulus differed. The described cascades provide leads for the development of new pharmacological and rehabilitative therapies to halt the progression of DDD.

## Introduction

Lumbar intervertebral disc (IVD) degeneration is a dominant factor in the etiology of low back pain (LBP) [1]. Disc degeneration is an age-related process [2], and may arise from any of several pathological conditions, such as trauma to the spine [3] or an inflammatory response. It is influenced by many factors, such as genetics [4], [5], systemic disorders (atherosclerosis, high cholesterol and diabetes) [6] and nutrient supply to the disc [7]–[9]. Mechanical (over)loading has been identified as a major extrinsic component in the onset and progression of IVD degeneration [10], [11].

The main function of the IVD is to transfer high magnitude axial forces, while maintaining flexibility of the spine. Loading is therefore a natural stimulus for the IVD and is even thought to be essential for maintenance of cell viability and matrix biology [12]. Conversely, excessive mechanical loading evokes catabolic cellular behavior, which may trigger a cascade towards disc degeneration, i.e. loss of proteoglycans and water from the disc, with subsequent changes in mechanical properties of the disc and further matrix breakdown [13]. Whether mechanical loading is a positive stimulus or induces damage to the IVD, is dependent on the type of load applied, its magnitude, duration and frequency [14], [15].

Furthermore, it has been reported that threshold values for beneficial or detrimental effects of static and dynamic loading differ between disc regions. In a study by Korecki et al. from 2008, dynamic overloading on bovine caudal discs caused an anabolic effect in the annulus and a catabolic effect in the nucleus [16]. Others report that static overloading causes cell death and disorganization of the matrix mostly in the annulus [17] and increased remodeling activity with up-regulation of collagen type 1 and MMP13 gene expression in the nucleus. Studies directly comparing static versus dynamic overloading, report that gene expression of collagen types 1 and 2 are downregulated in the annulus and upregulated in the nucleus with static compression, whereas dynamic overloading caused an up-regulation of these genes in both regions [18], [19]. A recent review by Chan et al. provides an excellent overview of the various studies reporting on the effects of loading on IVDs and their reported differences. From this overview, it becomes clear that experiments were only conducted on cell culture, tissue samples, IVDs of young, small animals or caudal discs [20]. It is important to realize that human lumbar discs and models used in these studies are disparate with regard to several aspects (e.g. size, biological age, notochordal status, lumbar vs. caudal, etc). These differences make reported catabolic or degenerative effects of (over)loading difficult to translate to the human disc [21]. Moreover, although these studies provide fundamental knowledge on several separate aspects of the response of IVDs to overloading, they do not provide an integral picture of mechanobiological effects of overloading on the IVD.

Mechanical loading, geometry, biomechanical properties and matrix content of caprine lumbar IVDs are highly comparable to human IVDs than discs from small animals, tail discs or discs taken from pigs [22]–[24]. In addition, like human IVDs, adult caprine IVDs lack notochordal cells after maturation [25]–[27]. Because the lumbar discs of the adult goat are fairly large (approximately 4–5 cm^2^ across and 4 mm height), they are more suitable for studying various parameters simultaneously on a single disc, when compared to small animal models. Also, processes of degeneration can be studied both in vitro and in vivo with the previously described in vivo goat IVD degeneration model [28], [29].

The purpose of the current study was to investigate whether dynamic and static overloading induce degenerative changes to caprine lumbar discs. We want to know how the biomechanical response changes over time, how this is connected to cell response, and what the effects are on the matrix. We have developed an ex vivo bioreactor, the Loaded Disc Culture System (LDCS), designed for whole organ culture of large species IVDs. The LDCS allows studies on IVD cells in their native environment and enables close monitoring and control of oxygen- and nutrient supply as well as mechanical loading conditions. The LDCS is capable of delivering both static and dynamic loads varying from 0 to 2 MPa in various frequencies, with continuous measurements of mechanical parameters. In a recent study we showed that we can maintain baseline properties of caprine IVDs in the ex vivo LDCS model for up to three weeks when applying simulated physiological loading (SPL) [29].

We aim to improve our understanding of the mechanobiology involved in load-induced IVD degeneration and thereby provide more insight in the early degenerative process. We hypothesize that both static and dynamic overloading lead to disc degeneration, resulting in changes in the biomechanical behavior of the discs cell survival, gene expression, and matrix structure and content. In addition, we expect that nucleus and annulus of the IVD respond differently to the two types of overloading. Ultimately, we aim to establish an ex vivo degeneration model that can be used as a reliable platform for pre- in vivo testing of novel interventions against disc degeneration.

## Methods

### IVD specimens and culture

A total of thirty-two lumbar spines from skeletally mature (3–5 year-old) Dutch milk goats were used for the experiments. Goat spines can be easily obtained from abattoirs in The Netherlands and as we do not need life animals for experimental testing, no approval of an ethical committee is required. Within 3 hours after slaughter, lumbar IVDs with adjacent cartilaginous endplates (Th13-L6) were dissected under sterile conditions using an oscillating surgical saw. The discs are dissected by sawing in two parallel planes as close as possible to the proximal and distal endplates. The sawing planes are perpendicular to the central axis through the IVD of the individual motion segment. IVDs were cleaned with sterile gauze to remove any debris, blood and excess muscle or ligament and placed in a 6-wells plate with culture medium prior to placement in the LDCS. From each spine, 2 IVDs (Th13-L1 and L5-L6) were used as baseline reference (day 0) for the parameters measured. The remaining IVDs were cultured for 7, 14, or 21 days in individual culture chambers in the previously described Loaded Disc Culture System (LDCS) [29], which is housed in an incubator at 37°C, 95% humidity, and 5% CO_2_. Discs were cultured in standard Dulbecco's Modified Eagle medium (DMEM; Gibco, Paisley, UK) with 10% fetal bovine serum (FBS, Hyclone, Logan, UT), 4.5 gr/L glucose (Merck KGaA, Darmstadt, Germany), 50 µg/ml ascorbic acid-2-phosphate (Sigma Aldrich, St. Louis, MO), 25 mmol/L HEPES buffer (Invitrogen, Merelbeke, Belgium), 10,000 µ/ml penicillin, 250 µg/L streptomycin, 50 µgr/mL gentamicin and 1.5 µgr/mL amphoterizin B (all from Gibco). Medium (40 ml per culture chamber) circulated continuously (3 ml/h) using a peristaltic pump and was exchanged every 48 hours and checked for pH (7.2–7.4) and osmolarity (360–380 mOsm; measured by cryoscopy).

### Loading protocols

Mechanical loading of the IVDs was strictly axial. Loading magnitudes and frequency were derived from *in vivo* pressure measurements in a lumbar segment of a goat during different activities (e.g. lying down, walking and jumping on a haystack) [30]. For standardization, all discs were subjected to a preload (Low Dynamic Loading (LDL); sinusoidal; 0.1–0.2 MPa; 1 Hz) during the first 8 hours of culture and all regimes ended again with 8 hours of LDL.

**Figure 1:**
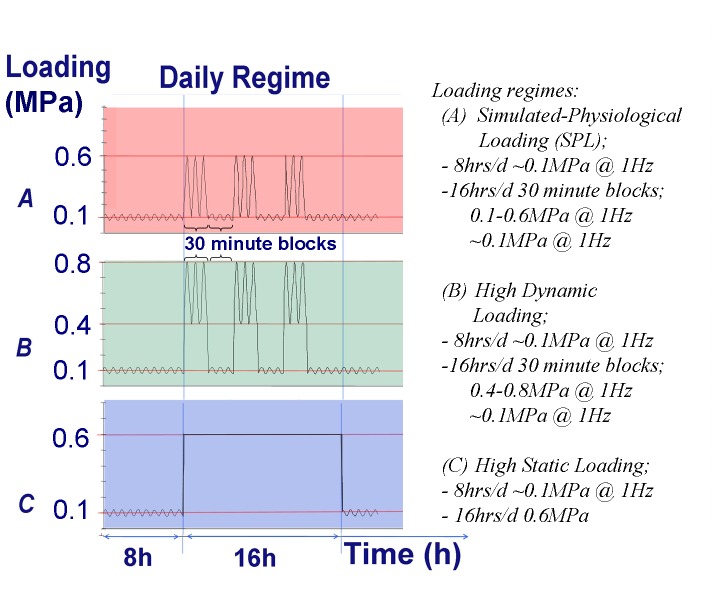
Scheme of the three daily loading regimes. Shown on the Y-axis is the axial load (MPa) as applied on the IVDs. Shown in the upper panel (red) is the simulated-physiological loading regime, in the middle panel (green) the high dynamic loading regime, and the lower panel (blue) the high static loading regime. All regimes start with 8 hours of low dynamic load around 0.1 MPa, after which a 16 hour loading regime is applied as indicated in the caption.

Discs were assigned to one of three loading groups (figure 1): simulated-physiological loading (SPL; sinusoidal load (1 Hz) alternating in magnitude every 30 minutes (∼0.1 MPa and 0.1–0.6 MPa) for 16 hours per day, followed by 8 hours of LDL); high dynamic loading (sinusoidal load (1 Hz) alternating in magnitude every 30 minutes ((∼0.1 MPa and 0.4–0.8 MPa for 16 hours per day, followed by 8 hours of LDL); or high static loading (static load of 0.6 MPa during 16 hours/day, followed by 8 hours of LDL).

The simulated-physiological loading regime (SPL) is comparative to activities such as lying down and walking in goats and relaxed standing and unsupported sitting in humans and has been shown to maintain native caprine disc properties over 21 days in LDCS culture [29]. The high dynamic loading regime pressures simulate jumping on a haystack in goats or lifting activity in humans [31], [32]. The high static loading has the same load dose as the high dynamic regime, yet applied to the disc in a constant static pressure. We hypothesis that these high loading regimes, simulating more strenuous activities for 16 hours per day will affect the caprine IVDs differently than the SPL loading. Also, the two represent different extremes; the high dynamic regime has a large dynamic displacement, whereas the static regime prolonged creep effect.

### Biomechanical assessment

Axial deformation was measured over the entire culture period. Overall axial subsidence (i.e. long term behavior) over the culture period was evaluated by looking at the deformation at the end of each daily loading phase. To assess the response of IVDs to daily loading and unloading we fitted a stretched exponential function [33], [34] to the deformation curves during loading and recovery phases measured at days 1, 7, 14 and 21. Parameters describing the fitted curves allow quantitative comparison between loading regimes, and to discern changes in deformation behavior of a loading regime over the culture period. The stretched exponential function is described by:
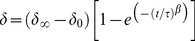
(1)


In this function δ (delta) is the displacement of the IVD at time t; τ (tau) is a time constant and β (beta) is a stretch constant. δ∞ (displacement at t  =  infinite) minus δ0 (displacement at the onset of the phase) equals the estimated total deformation (if the (un)loading phase would be infinitely sustained). τ represents the time required to reach 63% of the asymptotic value after the onset of the loading phase. β modulates the deviation from the standard exponential function, such that for β<1, deformation is faster-than-exponential for t<τ.

From the fitted curves, we also calculated the slopes (displacement per time) during the first and last hour of each load- and recovery phase. These slopes express the speed of deformation during the onset and end of the load and recovery phases, respectively.

### Histology and quantitative cell biology

Directly after dissection from the spine (baseline control) or after culture in the LDCS (day 7, 14 and 21), selected IVDs were fixated (4% formaldehyde) and decalcified (Kristensen's fluid). Paramidsagittal tissue slices (3 mm thick) were cut from the IVD specimen with a scalpel and embedded in paraffin. With a microtome, 3 micrometer ( µm) thin sections were cut and stained with either safranin-O (for proteoglycans) or Masson's trichrome (for collagen).

Cell viability was assessed in the nucleus pulposus (NP), and the inner (iAF) and outer annulus fibrosis (oAF). After removal of one endplate, selected IVDs (n≥6 for each group and time point) were incubated in a 6-well plate in serum-free medium containing 2 µM Celltracker Green (Chloromethylfluorescein, Molecular Probes, Eugene, OR) and 2 µM propidium iodide (Sigma) under free-swelling conditions. After 1 hour, IVDs were washed in PBS three times and flash-frozen. 10 µm transverse cryosections were cut with a cryostat. Images (1048×1342 pixels) were taken at 10× magnification (surface area ≈1 mm2) using fluorescent light on an inverted microscope (Leica DM6000, Wetzlar, Germany; filters: I3 S450–490 nm and N2.1 S515–560 nm). The total number of cells per area (cell density) and the percentage of live cells (100% (# live cells/# total cells)) were determined using 10 images per region for each IVD. Co-labelled cells were counted for the cell density measurement, but were excluded from the analysis of cell viability. A fresh (day 0) IVD was used as positive control. As a negative control, a thoracic IVD, which underwent a freeze-thawing cycle three times prior to staining, was used.

### RNA isolation, cDNA synthesis and RT-qPCR

Directly after culture (at 7, 14 or 21 days), IVDs were dissected and a half nucleus and outer annulus (n≥8 for each region, experimental group and time point) were placed in special 2 ml tubes (MagnaLyser Green beads) containing ceramic beads and lysis solution and homogenized using a automated shaker (MagnaLyser) with 4 runs of 30 seconds at 6500 rpm with in-between cooling and stored at −80°C for further processing. Total RNA was isolated with the MagnaPure robot using the RNA isolation kit III (all from Roche Diagnostics, Almere, Nederland). cDNA synthesis was performed using Superscript Vilo® (Invitrogen) and real-time PCR reactions on cDNA samples (triplo) were performed using the SYBRGreen reaction kit (Roche Diagnostics) both according to the manufacturer's instructions in a LightCycler 480 (Roche Diagnostics). All samples for each time-point, were unique and derived from the nucleus or annulus of a single intervertebral disc. All cDNA samples from the nucleus or annulus were quantified in the same PCR run using the 384 well plate system (Roche Diagnostics). IVD cell gene expression was assessed for seven anabolic genes; collagen types 1, 2 and 6, lysyl oxidase (LOX), procollagen-lysine,2-oxoglutarate 5-dioxygenase 3 (PLOD 3), aggrecan and biglycan, seven catabolic/remodelling genes; TIMP (tissue inhibitors of metalloproteinase) 1, 2 and 3, MMP (matrix metalloproteinase) 13 and 14, ADAMTS (a disintegrin and metalloproteinase with thrombospondin motifs) 4 (Ad4) and 5 (Ad5), and seven inflammatory-related genes C-JUN, BIP (heat shock protein A5; HSPA5), cyclooxygenase 2 (COX-2) and IL (interleukin) 1, 6, 8 and 10. The primers used for the gene expression analyses are shown in table 1. Expression of two housekeeping genes was quantified: YWHAZ (tyrosine 3-monooxygenase/tryptophan 5-monooxygenase activation protein) and 18S (ribosomal RNA). The stability of expression of the housekeeping genes was analyzed per cDNA sample using geNorm software (http://medgen.ugent.be/~jvdesomp/genorm/). The expression of all genes was in the range of YWHAZ expression levels, therefore this housekeeping gene was used as normalization factor. Relative gene expression is shown as the ratio between the expression of the gene of interest divided by YWHAZ expression of the same sample. No technical replicates were included in the statistical analyses. Samples with no detectable RNA concentration of the target gene, but with detectable gene concentration of the housekeeping genes (Ct<18) were assigned a Ct of 45 (i.e. detection threshold).

**Table 1:** Primer sequences used for PCR.

Target gene		Oligonucleotide sequence	Annealing temperature (°C)	Product size (bp)
18S	Forward	5' GTAACCCGTTGAACCCCATT 3'	57	151
	Reverse	5' CCATCCAATCGGTAGTAGCG 3'		
YWHAZ	Forward	5' GATGAAGCCATTGCTGAACTTG 3'	56	229
	Reverse	5' CTATTTGTGGGACAGCATGGA 3'		
Collagen 1a1	Forward	5' TCCAACGAGATCGAGATCC 3'	57	191
	Reverse	5' AAGCCGAATTCCTGGTCT 3'		
Collagen 2a1	Forward	5' TGTCAGGGCCAGGATGT 3'	56	256
	Reverse	5' CTCCTTTCTGTCCCTTTGG 3'		
Collagen 6	Forward	5' CAGTGACGAGGTGGAGATCAT 3'	57	294
	Reverse	5' ATGGCCACCGAGAAGAC 3'		
Aggrecan	Forward	5' CAACTACCCGGCCATCC 3'	57	160
	Reverse	5' GATGGCTCTGTAATGGAACAC 3'		
Biglycan	Forward	5' TACAGCGCCATGTGTCCTT 3'	59	274
	Reverse	5' GGTGGTTCTTGGAGATGTAGAG 3'		
LOX	Forward	5' TGGGCTCACAGTACCAG 3'	57	209
	Reverse	5' GTAGCCAGCTTGGAACC 3'		
PLOD3	Forward	5' CTGTGGCTTCTGCAACCAGG 3'	57	346
	Reverse	5' GGCGTCCAGGCTGAAGTAGA 3'		
TIMP1	Forward	5' CACAGACGGCCTTCTGCAA 3'	57	211
	Reverse	5' TTGTGGGACCTGTGGAAGT 3'		
TIMP2	Forward	5' CTGAACCACAGGTACCAGAT 3'	57	237
	Reverse	5' TGCTTATGGGTCCTCGATG 3'		
TIMP3	Forward	5' AGGACGCCTTCTGCAACTC 3'	57	163
	Reverse	5' GCTTCCGTATGGATGTACTG 3'		
MMP13	Forward	5' GGAGCATGGCGACTTCTAC 3'	56	208
	Reverse	5' GAGTGCTCCAGGGTCCTT 3'		
MMP14	Forward	5' CTGAGATCAAGGCCAATGTTC 3'	56	206
	Reverse	5' CTCACGGATGTAGGCATAGG 3'		
ADAMTS4	Forward	5' CATCCTACGCCGGAAGAGTC 3'	57	278
	Reverse	5' GGATCACTAGCCGAGTCACCA 3'		
ADAMTS5	Forward	5' GTGGAGGAGGAGTGCAGTTTG 3'	57	320
	Reverse	5' TTCAGTGCCATCGGTCACCTT 3'		
c-JUN	Forward	5' GGATCAAGGCGGAGAGGAA 3'	57	232
	Reverse	5' TGCAACTGCTGCGTTAGCAT 3'		
BIP (HSPA5)	Forward	5' TGCCTACCAAGAAGTCTCAGAT 3'	55	214
	Reverse	5' TCAGCTGTCACTCGAAGAAT 3'		
COX2	Forward	5' AGACCAGGCACCAGACCAAAGA 3'	56	299
	Reverse	5' GCATTCTTTGCCCAGCACTT 3'		
IL1	Forward	5' TGGAGCAACAAGTGGTGTTCT 3'	57	270
	Reverse	5' GAGAGGTGCTGATGTACCAGTT 3'		
IL6	Forward	5' CTCTTCACAAGCGCCTTCAGT 3'	57	248
	Reverse	5' GCCAGTGTCTCCTTGCTGTT 3'		
IL8	Forward	5' TCTGCAGCTCTGTGTGAAG 3'	57	147
	Reverse	5' TGTGTTGGCGCAGTGTGG 3'		
IL10	Forward	5' GGGTTGCCAAGCCTTGTC 3'	57	185
	Reverse	5' CCACGGCCTTGCTCTTGTT 3'		

List of forward and reverse primers used for the gene expression analyses showing the oligonucleotide sequences, annealing temperature and product size.

### Quantitative biochemistry

Tissue samples were taken from nucleus, inner annulus (iAF) and outer annulus (oAF) regions of the IVDs (n≥12 for each region, group and time point). Water content of each sample was calculated from measured wet (ww) and dry weights (dw), before and after freeze drying (speedvac). Dry weight samples (≈1 mg) were digested in a papain-digestion (5 mmol/L L-cysteine, 50 mmol/L EDTA, 0.1 M sodium acetate, pH titrated to 5.53 using 1 M NaOH and 3% (v/v) papain) at 65°C. Papain-digestion suspension (10 µL) was analyzed using a 1.9-dimethyl-methylene blue (DMMB) assay (Biocolor Ltd., Carrickfergus, UK) in accordance with the manufacturer's description and measuring light absorption of samples with a spectrophotometer at a wavelength of 656 nm. Measured amount of glycosaminoglycans (GAG) for each sample was normalized to tissue dry weight. From the remaining papain-digestion solution, 500 µL was used for analysis of hydroxyproline (Hyp) as a measure of total collagen content. Digestion samples were hydrolyzed in 6 M HCl at 105°C to release hydroxyprolines. A dimethylamino-benzaldehyde (DMBA) assay was used to assess Hyp content in the solutions, measuring the absorption of the samples at a wavelength of 570 nm. A hydroxyproline calibration curve made with a standard solution (60 µg/ml hydroxyproline) was used to quantify sample content. Total collagen was expressed as micrograms hydroxyproline per milligram tissue dry weight [35], [36].

### Statistical analysis

All data was analyzed using linear mixed models. Separate analyses were performed for the three disc regions. Experimental outcome parameters were included as dependent variables in the models. The models included a fixed effect for test duration (for cell viability and cell density, water, GAG en collagen) or experimental loading condition (for gene expression). A random effect for each goat (n = 30, for gene expression, water, GAG and collagen) or goat and IVD combination (for cell viability and cell density) was included in the model. The random effect was needed to account for correlation of measurements between the multiple discs from a single goat. Mean outcomes for test duration were compared within each loading group. The same set of day 0 measurements were used as baseline measurements for all loading groups. Additional mixed models were fitted to compare the mean outcomes between loading groups for each test duration separately. Bonferroni posthoc testing was used to compare means for the different test durations with baseline (first set of mixed models) and means between different loading groups (additional set of mixed models).

## Results

### Biomechanical parameters

**Figure 2:**
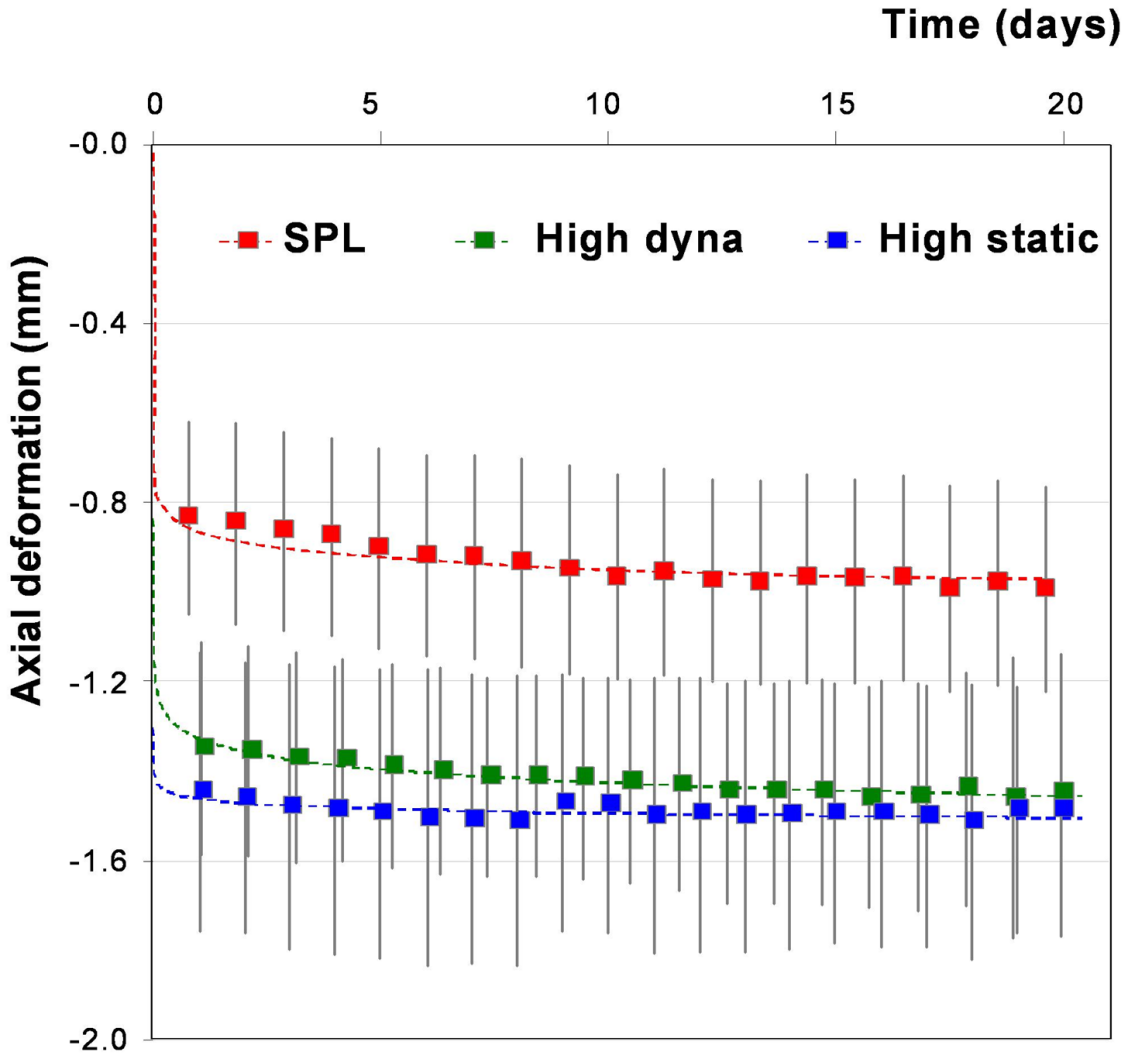
Overall subsidence (mean IVD height loss ± SD) of IVDs during the culture period. All IVDs show a settling effect in the first period of culture and a more gradual subsidence over the entire culture period. Total subsidence is smallest in the SPL loaded group (red), and much higher in IVDs subjected to high dynamic loading (green) and high static loading (blue). Dynamically loaded IVDs show a more gradual course of subsidence over the culture period, whereas subsidence in the high static load group (blue) is largest and occurs mostly in the first days of culture.

**Figure 3:**
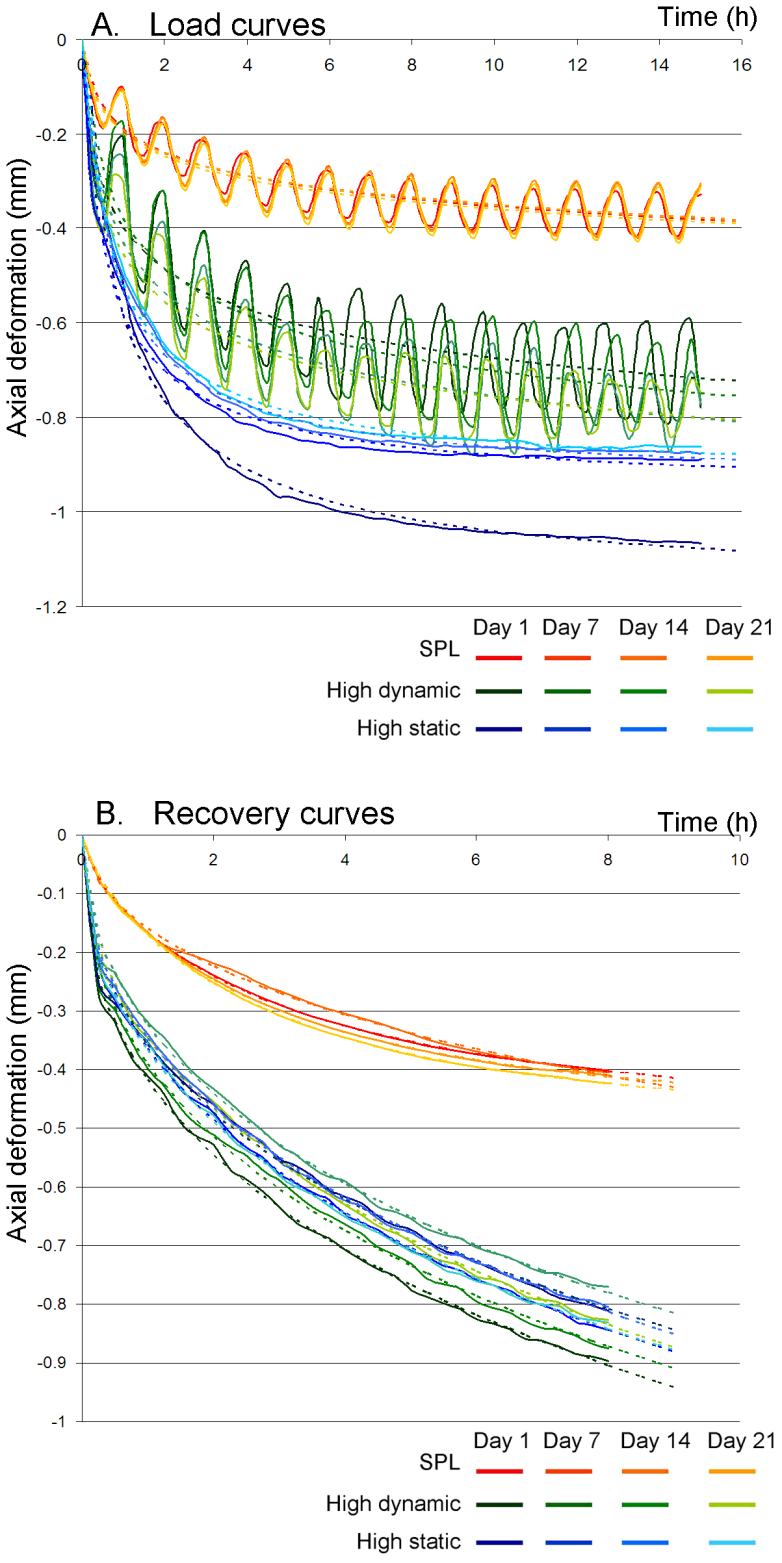
a. Mean axial deformation (mm) curve during the loading phase. Shown are the SPL (red), high dynamic (green) and high static (blue) loading regime at 1, 7, 14 and 21 days (respectively dark to lighter colored lines) of culture. The dotted lines represent the stretched exponential functions fitted to the deformation curves. b. Mean axial deformation (mm) curve during recovery phase. Shown are the SPL (red), high dynamic (green) and high static (blue) loading regime at 1, 7, 14 and 21 days (respectively dark to lighter colored lines) of culture. The dotted lines represent the stretched exponential functions fitted to the deformation curves.

Deformation behavior was rather consistent for all groups throughout the culture period, although discs showed an average overall subsidence of around 0.10 mm between days 1 and 21 (table 2, figure 2). The overall subsidence was smallest and most gradual for the SPL (red), and highest and least gradual for the high static load (blue). Daily axial deformation patterns during loading (figure 3a) and recovery phases (figure 3b) differed between loading regimes. High static load caused a faster loading response than the other regimes. This results in smaller values for tau and beta (table 2). Total axial deformation during the loading phase was highest in the high static (blue), intermediate in the high dynamic (green), and smallest in the SPL regime (red-yellow, figure 3a). In addition, in the high static loading group, most subsidence occurred directly during day one, after which deformation and recovery behavior remained relatively stable. Displacement curves during loading were rather constant in the SPL and high dynamic loading groups. In all loading regimes subsidence under loading was a faster process than height gain during recovery. However, the speed of the recovery process depended on the loading (figure 3b). In the high dynamic loading group, recovery declined over the culture period, which causes the gradual overall subsidence. For both overloading regimes, recovery was far from completed after 8 hours, indicated by the estimated values of tau, which exceed 8 hours in all cases, and the end slope which deviates considerably from 0 (table 2).

**Table 2:** Descriptive parameters of axial deformation curves of IVDs during the loading- and recovery phases.

Phase	Load	Day	Tau		Beta		Deformation (mm)		Start slope (mm/hrs)		End slope (mm/hrs)	
Load	SPL	1	3.4	±0.8	1.06	±0.18	0.33	±0.08	0.20	±0.05	0.007	±0.001
		7	3.0	±0.7	1.08	±0.11	0.31	±0.07	0.21	±0.05	0.006	±0.002
		14	2.9	±0.4	1.10	±0.11	0.31	±0.07	0.21	±0.04	0.006	±0.001
		21	2.8	±0.4	1.08	±0.11	0.31	±0.07	0.22	±0.04	0.006	±0.001
	High	1	3.4	±0.4	1.45	±0.35	0.77	±0.08	0.42	±0.04	0.011	±0.003
	dyna	7	3.0	±0.2	1.27	±0.21	0.77	±0.08	0.42	±0.10	0.013	±0.008
		14	3.0	±0.4	1.23	±0.32	0.77	±0.21	0.46	±0.06	0.013	±0.008
		21	2.8	±0.3	1.38	±0.42	0.75	±0.25	0.48	±0.06	0.012	±0.007
	High	1	1.5	±0.6	0.77	±0.10	1.07	±0.31	0.65	±0.21	0.017	±0.008
	static	7	1.8	±0.7	0.94	±0.06	0.89	±0.16	0.61	±0.12	0.010	±0.003
		14	1.3	±0.6	0.81	±0.25	0.88	±0.17	0.58	±0.14	0.012	±0.004
		21	1.4	±0.5	0.83	±0.22	0.86	±0.15	0.56	±0.12	0.012	±0.004

Phase	Load	Day	Tau		Beta		Deformation (mm)		Start slope (mm/hrs)			
Recovery	SPL	1	3.5	±0.8	0.72	±0.10	0.40	±0.09	0.17	±0.05	0.014	±0.004
		7	11.2	±5.6	0.62	±0.08	0.40	±0.07	0.16	±0.03	0.020	±0.005
		14	3.0	±0.7	0.76	±0.10	0.41	±0.07	0.17	±0.02	0.013	±0.005
		21	3.0	±0.8	0.77	±0.09	0.42	±0.07	0.17	±0.02	0.013	±0.005
	High	1	13.7	±4.5	0.50	±0.04	0.90	±0.09	0.41	±0.03	0.038	±0.007
	dyna	7	15.0	±2.8	0.51	±0.06	0.88	±0.05	0.39	±0.05	0.039	±0.007
		14	10.9	±6.0	0.66	±0.23	0.77	±0.14	0.32	±0.09	0.033	±0.011
		21	12.8	±5.7	0.62	±0.16	0.83	±0.14	0.34	±0.08	0.038	±0.010
	High	1	14.5	±4.0	0.55	±0.11	0.81	±0.17	0.35	±0.10	0.037	±0.007
	static	7	13.0	±5.6	0.56	±0.09	0.84	±0.20	0.36	±0.10	0.037	±0.012
		14	12.9	±5.8	0.57	±0.09	0.81	±0.20	0.34	±0.10	0.037	±0.013
		21	11.9	±5.8	0.56	±0.08	0.83	±0.15	0.36	±0.08	0.036	±0.012

Descriptive parameters (means ± SD) of the axial deformation of IVDs during the load and recovery phase of the SPL, high dynamic and high static regime on day 1, 7, 14 and 21. Tau (time constant) and beta (stretch constant) are derived from a stretch exponential fit to the deformation curves. Deformation (difference in IVD height at start and end of curve; mm) and the start (first hour) and end (last hour) slope (speed of axial deformation; mm/hrs) were calculated from the fitted curve data.

### Histology

**Figure 4:**
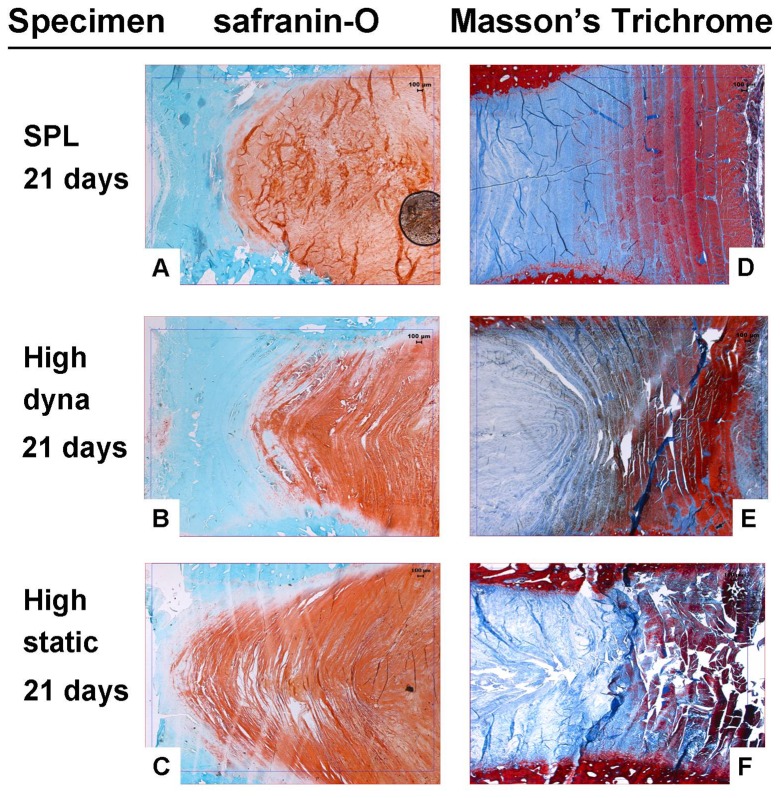
Histological sections. Representative images (2,5× magnification) of midsagittal sections of IVD specimens after 21 days of culture under SPL (upper row), high dynamic (middle) and high static (lower) loading conditions. The left column shows the transitional zone between the nucleus and posterior annulus region (posterior annulus facing left) in Safranin-O stained sections. In the right column the anterior annulus (inner annulus left, outer annulus right) of the IVDs is shown in sections stained with Masson's trichrome.

Typical examples of histological images are shown in figure 4. Safranin-O stained sections (figure 4. A,B,C) show the transitional zone between the nucleus (red) and posterior annulus (blue). IVDs subjected to the high loading regimes show a gradual displacement and blurring of the transition zone, and disruption of the structure of both nucleus and annulus (B and C) when compared to IVDs subjected to SPL (A).

Masson's trichrome stained sections (figure 4. D,E,F) clearly show bulging of nucleus tissue against the anterior inner-annulus (outer-anterior side facing right) in high dynamically loaded discs (E) and disruption of the annulus in the midline of high statically loaded discs (F). In some discs this could already be observed after 7 or 14 days of culture.

### Cell viability and density

**Figure 5:**
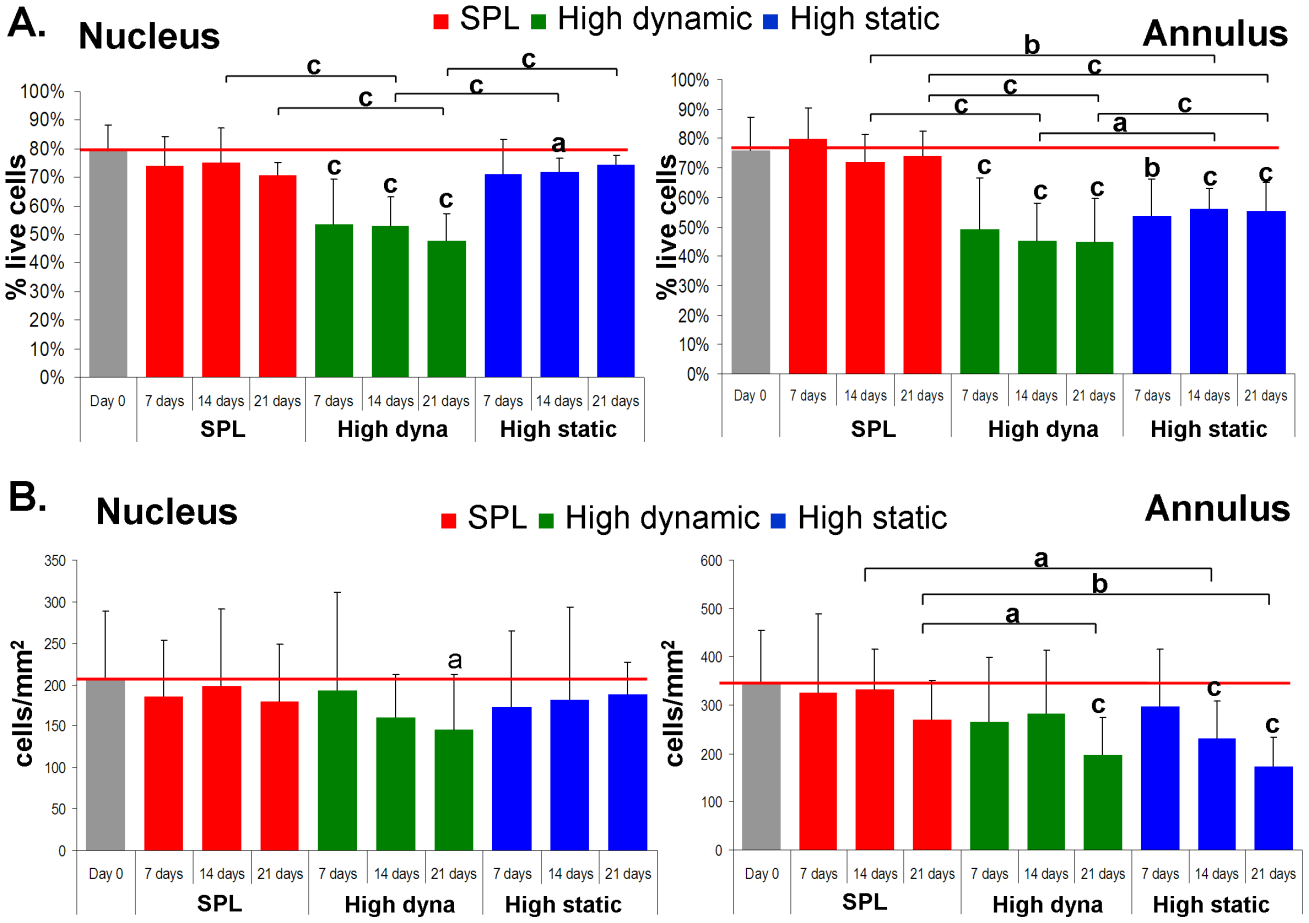
a. Cell viability. Shown is the cell viability (mean percentage live cells +SD) per experimental group and test duration. The left graph shows the viability in the nucleus region and the right graph the viability in the outer annulus. *P* value letters on top of bars indicate statistical difference with baseline (day 0) and brackets indicate significant differences between groups when comparing in a linear mixed model with Bonferroni post-hoc testing: *P* values are indicated by: ^a^ *p*<0.05; ^b^ *p*<0.01; ^c^ *p*<0.001. b. Cell density. Shown is the cell density (mean cell count/mm2 +SD) per experimental group and test duration. The left graph shows the viability in the nucleus region and the right graph the viability in the outer annulus. *P* value letters on top of bars indicate statistical difference with baseline (day 0) and brackets indicate significant differences between groups when comparing in a linear mixed model with Bonferroni post-hoc testing: *P* values are indicated by: ^a^ *p*<0.05; ^b^ *p*<0.01; ^c^ *p*<0.001.

Average cell viability (±SD) at baseline (day 0) in all regions of the disc was between 70–80% (NP: 79.3±9.2%; iAF: 72.0±10.3%; oAF: 75.9±11.3%). Cell density was lowest in the nucleus (206.7±81.8 cells/mm^2^), intermediate in the inner annulus (291.4±116.0 cells/mm^2^) and highest in the outer annulus (344.3±110.6 cells/mm^2^). Cell viability in the SPL group did not differ significantly from baseline at any time-point (figure 5a). Cell density in the outer annulus was reduced to 270.8 (±80.4) cells/mm^2^ after 21 days of culture with SPL, however, compared to baseline this difference was not significant (figure 5b).

After 7 days of culture with high dynamic loading, cell viability dropped significantly in all three regions of the IVD, to respectively 53.7% in the nucleus (*p*<0.001^a^), 50.6% in the inner annulus (*p = *0.001^a^; *p* = 0.018^b^) and 49.1% in the outer annulus (*p*<0.001^a^; *p* = 0.051^b^). After 21 days, cell viability was reduced to 47.9% in the nucleus (*p*<0.001^a,b^), 49.7% in the inner annulus (*p*<0.001^a,b^) and 44.6% in the outer annulus (*p*<0.001^a,b^). Cell density was significantly reduced in nucleus and outer annulus at day 21 (resp. 145.0 cells/mm^2^ (*p*<0.001^a^) and 197.9 cells/mm^2^ (*p*<0.001^a^; *p* = 0.037^b^)).

The high static load group showed a different pattern in the loss of cell viability and cell density. In the nucleus, cell viability decreased only slightly at all three time-points, with differences reaching significance only at day 14 (72.0%; *p* = 0.012^a^). In the outer annulus, cell death was more pronounced. Cell viability declined to 53.8% (*p* = 0.004^a^) already after 7 days and stabilizing thereafter. The total number of cells in the outer annulus decreased steeply at every time point. Cell density dropped to 231.1 cells/mm^2^ at days 14 (*p*<0.001^a^; *p* = 0.017^b^) and 174.6 cells/mm^2^ at day 21 (*p*<0.001^a^; *p* = 0.005^b^).

### Gene expression

**Figure 6:**
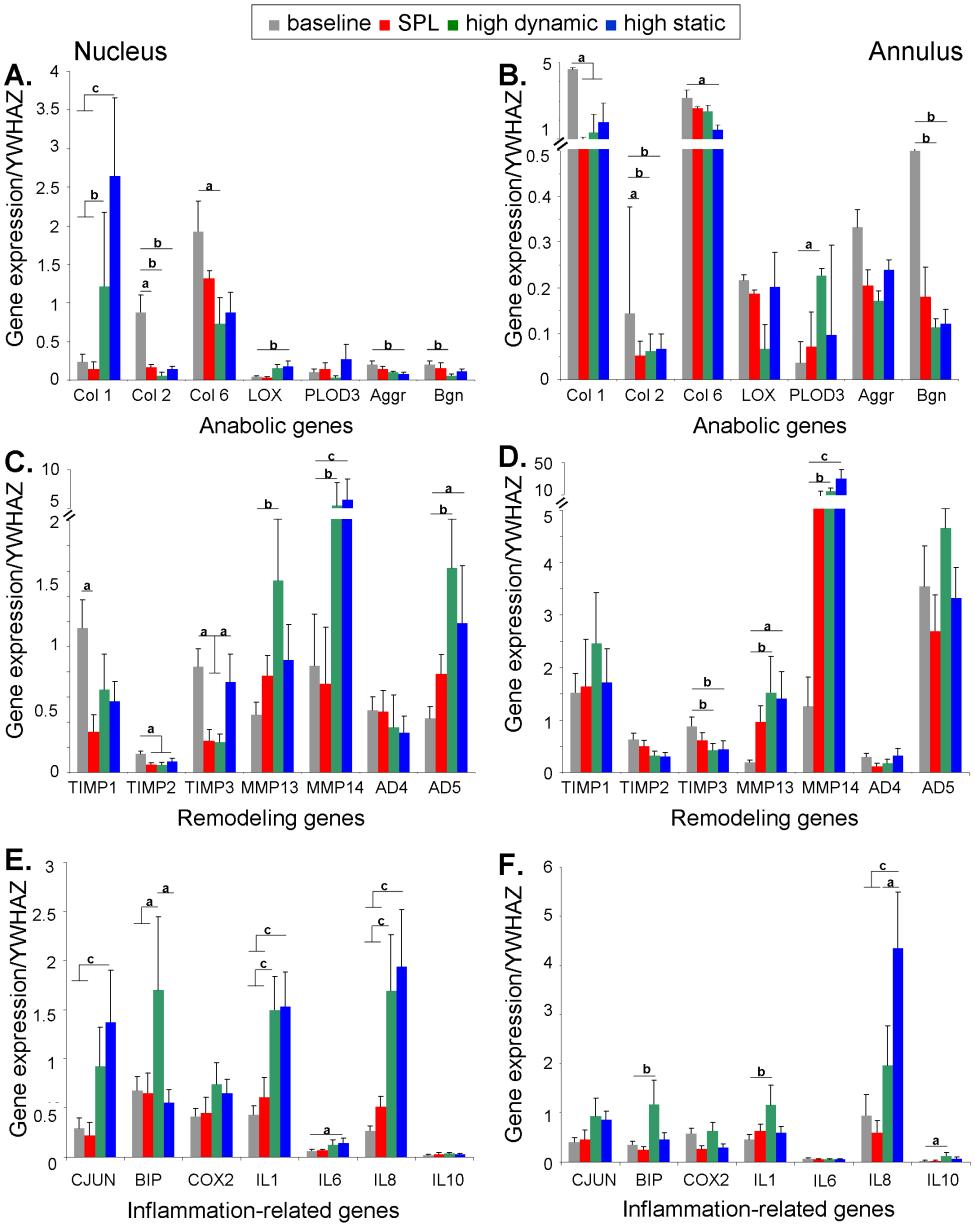
Relative gene expression. Shown is the gene expression relative to YWHAZ (log means +SEM) in the baseline, SPL, high dynamic and high static group. The left column includes the data for the nucleus region and in the right column for the outer annulus region. Graph rows from top to bottom show respectively the anabolic, remodeling and inflammation-related genes. Brackets indicate significant statistical differences between groups when comparing in a linear mixed model with Bonferroni post-hoc testing. *P* values are indicated by: ^a^ *p*<0.05; ^b^ *p*<0.01; ^c^ *p*<0.001.

Gene expression patterns of IVD samples depended strongly on loading regime (table 3, figure 6). Within load groups no significant effect of culture duration was observed. For this reason, it was decided to compare the mean expression levels of each gene at day 0 with the average mean expression levels for this gene during culture, i.e. gene expression averaged over days 7, 14 and 21. For these analyses time was dichotomized; the first category corresponded to the day 0 measurements and the second category to measurements made on day 7, 14 and 21.

**Table 3:** Qualitative assessment of relative gene expressions; *p*-value differences of experimental groups compared to baseline.

		Nucleus			Annulus	
	SPL	high dyna	high static	SPL	high dyna	high static
Col1	=	↑↑	↑↑↑	↓	↓	↓
Col2	↓	↓↓	↓↓	↓	↓↓	↓↓
Col6	=	↓	=	=	=	↓
LOX	=	=	↑↑	=	=	=
PLOD3	=	=	=	=	↑	=
Aggrecan	=	=	↓↓	=	=	=
Biglycan	=	↓↓	=	=	↓↓	↓↓
TIMP1	↓	=	=	=	=	=
TIMP2	↓	↓	↓	=	=	=
TIMP3	↓	↓	=	=	↓↓	↓↓
MMP13	=	↑↑	=	=	↑↑	↑
MMP14	=	↑↑	↑↑↑	=	↑↑	↑↑↑
ADAMTS4	=	=	=	=	=	=
ADAMTS5	=	↑↑	↑	=	=	=
CJUN	=	=	↑↑↑	=	=	=
BIP	=	↑	=	=	↑↑	=
COX2	=	=	=	=	=	=
IL1	=	↑↑↑	↑↑↑	=	↑↑	=
IL6	=	=	↑	=	=	=
IL8	=	↑↑↑	↑↑↑	=	=	↑↑↑
IL10	=	=	=	=	↑	=

Qualitative assessment of relative gene expressions data per disc region, experimental group and gene. Arrows represent *p*-value differences of experimental group gene expression compared to baseline gene expression. One arrow indicates *p*<0.05; two arrows indicates *p*<0.01; three arrows indicates *p*<0.001 when compared to baseline gene expression.

Of the anabolic genes, collagen type 1 was hardly expressed in the nucleus and highly expressed in the annulus at baseline (upper panel table 3 and figure 6a and 6b). In the high dynamic and static loaded groups collagen type 1 was highly up-regulated in the nucleus and down-regulated in the annulus. In the SPL group expression of collagen type 1 remained unchanged in the nucleus, but was down-regulated in the annulus. Collagen types 2 and 6 were strongly expressed in the nucleus at baseline. Collagen 2 was significantly down-regulated in both the nucleus and annulus regions in all loading groups, whereas collagen 6 was down-regulated in the nucleus with high dynamic loading and in the annulus with high static loading. Expression of LOX was up-regulated in the nucleus of the static group, whereas PLOD3 was up-regulated in the annulus of the high dynamic group. Aggrecan showed down-regulation in the nucleus in the static group and biglycan in the high dynamic group. In the annulus biglycan was down-regulated both in high dynamic and static loading.

With regard to the remodeling genes, the overall expression of TIMPs is reduced in nucleus and annulus regions for all load groups compared to baseline (table 3 middle panel and figure 6c and 6d). MMP 1 showed very low expression levels in all groups, without any significant changes (data not shown), whereas expression of MMP 13 and 14 were upregulated in both regions of the high load groups. ADAMTS 4 expression did not change significantly in the culture groups, whereas ADAMTS 5 was up-regulated in the nucleus for both high dynamic and static loading.

The overall expression of inflammation-related genes in the nucleus was up-regulated in both the high dynamic and static load groups, with c-JUN, IL 1 and 8 showing the largest changes (table 3 lower panel and figure 6e and 6f). In the annulus region changes were less pronounced; IL 1 and 10 expressions were significantly up-regulated with high dynamic loading and IL 8 with high static loading.

### Extracellular matrix content

**Figure 7:**
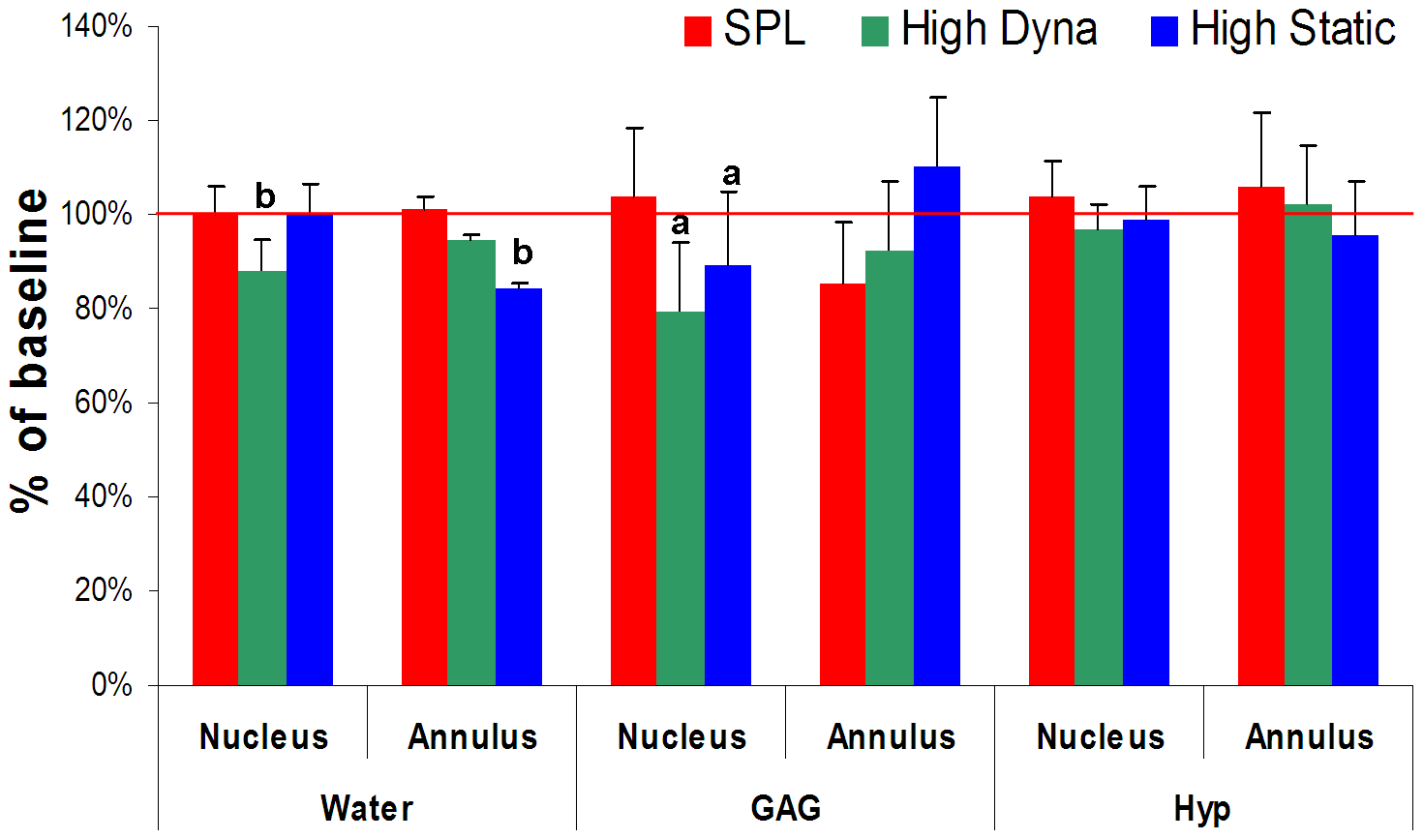
Extra-cellular matrix content. Relative water (mean percentage water +SD; left), GAG (mean GAG mg/dry weight +SD) and total collagen content (mean Hyp mg/dry weight +SD) for the SPL, high dynamic and high static group after 21 days of culture. Values are expressed as percentage of baseline (day 0). *P* value letters on top of bars indicate significant difference with baseline when comparing in a linear mixed model with Bonferroni post-hoc testing: *P* values are indicated by: ^a^ *p*<0.05; ^b^ *p*<0.01; ^c^ *p*<0.001.

In figure 7 all extracellular matrix parameters are shown per load and region at 21 days. All baseline (day 0) values for water, GAG and Hyp were set at 100% and changes after 21 days were expressed relative to these values. Water content (mean ±SD) of fresh IVDs at baseline was 72.9%±0.6 in the nucleus, 63.2%±0.6 in the inner annulus, and 56.9%±0.7 in the outer annulus. After 21 days water content in the nucleus was significantly lower in the high dynamic loading group (64.4%±0.8; *p* = 0.008), whereas water content in the outer annulus was reduced in the high static load group (48.0%±0.1; *p* = 0.014) relative to baseline (figure 7, left panel).

Glycosaminoglycan content (mean ±SD) of baseline samples was 444.3±92.6 µg GAG/mg dry weight (dw) in the nucleus, 231.0±83.9 µg GAG/mg dw in the inner annulus and 75.5±68.8 µg GAG/mg dw in the outer annulus. Only after 21 days we found a significant loss of GAG in the nucleus region with high dynamic loading (352.3±50.6 µg GAG/mg dw; *p* = 0.018) and high static loading (350.8±136.2 µg GAG/mg dw; *p* = 0.048) when compared to baseline (figure 7, centre panel).

Collagen content (mean ±SD) at baseline was 20.5±9.7 µg hyp/mg dw in the nucleus, 36.2±10.4 µg hyp/mg dw in the inner annulus and 64.0±18.6 µg hyp/mg dw in the outer annulus. No significant differences in measured mean hydroxyproline content were measured in any region at any time point when compared to baseline (figure 7, right panel).

## Discussion

We investigated the effects of high dynamic and static loading on large species IVDs in an ex-vivo culture model. We found that caprine lumbar discs respond differently to dynamic and static overloading with respect to biomechanical behavior, cell survival, gene expression and matrix content. The response to overloading in nucleus and annulus progressed in time and ranged from disc remodeling activity to early load-induced disc degeneration.

Due to the complex structure and composition of the IVD, its biomechanical behavior is highly non-linear and time dependent [33], [34]. Deformation in response to loading is a result of both viscoelastic and poroelastic processes [23], [37]. The instantaneous and fast responses to dynamic loading may be attributed primarily to deformation of the matrix structures and reflect the viscoelastic behavior of these structures, whereas the long term response (i.e. long term creep behavior) is most likely due to a transfer of fluids. In this study, we could clearly see that overall subsidence of the IVDs depended on the amount of loading that the IVD received (figure 2). IVDs subjected to the static loading regime received on average the highest loading, which was associated with the largest subsidence. Nevertheless, overall deformation was almost as large in the high dynamic regime, although the total average load is only 62% of the load in the high static regime. Height is regained by the relatively quick recoil of the matrix and the much slower recovery of fluids [38], [39]. For the high loading regimes, the 8 hour recovery time is too short to regain the water pressed out during the first loading cycle (figures 3a and 3b). One may therefore assume that these discs remain in a less hydrated state as compared to the IVDs in the SPL group. Even though the overall subsidence in both overloading regimes is similar, the way and intensity of loading of the matrix structure is very different (table 2). In the dynamic loading regime the matrix is incessantly in motion, whereas the static load provides a steady creep of the matrix.

These differences in biomechanical response have their impact on the histological as well as the cellular level. When studying the histological sections we could observe structural damage to the matrix in both nucleus and annulus inflicted by both overloading regimes. We observed differences in the onset as well as the pattern of damage throughout the disc between loading regimes. With high dynamic loading, all regions are moderately affected after 21 days, whereas with high static loading especially the outer annulus (both posterior and anterior) was damaged, in some cases already after 7 days of culture (figure 4). Analogous degenerative changes occurred at the cellular level. High dynamic loading caused substantial cell death within 7 days in all disc regions (figure 5a), with cell density dropping significantly after 21 days when comparing to baseline and SPL (figure 5b). The decrease in cell viability and density with high static loading was most pronounced in the annulus region. Mechanical loading experiments on IVDs, as well as FE analyses, have shown that especially during static loading of the intervertebral disc, there are high shear stresses on the annulus [40]–[43]. These shear stresses may be responsible for the strong drop in cell viability and cell density in the outer annulus of IVDs under high static loading. On the other hand, the nucleus was much more affected by high dynamic loading. Apparently, the nucleus is relatively insensitive to sustained high pressure, but less resistant to intense dynamic deformation. Loss of GAGs and water was also most pronounced in the nucleus after high dynamic loading. This corresponds with the decline in recovery over the culture period observed in the high dynamic loading group.

To ascertain which catabolic and inflammation-related regulatory genes may be responsible for these changes in water and GAG content, we studied a selection of anabolic, remodeling and inflammation-related genes that have been shown to be involved in load-induced matrix degradation of the IVD [44], [45]. The expression of anabolic genes was strongly affected by both high dynamic and static loading, especially in the nucleus. We found up-regulation of collagen type 1, LOX and PLOD 3 expression and down-regulation of collagen type 2, aggrecan and biglycan (figure 6, upper panel). This gene expression pattern corresponds well with the third phase of matrix turnover, reported by Antoniou et al. [46], which is described as a degenerative or fibrotic phase. In this line of thought, our results may be interpreted such that nucleus cells remodel their surrounding matrix in response to the loading stresses. Similar observations have been made in other load-induced degeneration studies [14], [47]–[49].

We measured a significant loss of GAG in the nucleus with high dynamic and static overloading (figure 7). Correspondingly, MMP 13 and 14 and ADAMTS 5 expression were up-regulated in the nucleus of IVDs in both overloading groups, whereas ADAMTS 4 expression did not change significantly (figure 6, middle panel). ADAMTS 5 has been identified as the major aggrecanase to be active with matrix remodeling in response to mechanically-induced joint injury [50] and ADAMTS 4 to be the pro-inflammatory cytokine-induced aggrecanase, active in OA type degeneration [51]. The observed ADAMTS 5 and inflammation-related gene expression (i.e. up-regulation of c-JUN, BIP, IL 1 and 8; figure 6, lower panel) in the current study are in accordance with these findings in the sense that they suggest mechanically induced disc degeneration.

High dynamic and high static loading influence the IVDs biomechanical properties and cell responses already within the first week of culture, but did not affect the matrix composition as promptly, as changes in ECM content relative to baseline reached significance only after 21 days of culture (i.e. loss of water and GAG) (figure 7). Cell stress, indicated by up-regulation of C-JUN, BIP and IL 1 expression without a significant loss in cell viability, seems correlated to matrix remodeling activity, exemplified by the up-regulation of MMP and ADAMTS 5 [52]. Cell death together with a progressive loss of cell density on the other hand, seems to be correlated with an absolute loss of matrix content. This interval between the response of the cells and the subsequent loss of matrix proteins due to the IVDs overloading, could be explained by the time that matrix remodeling enzymes, such as ADAMTS and MMPs, need to cleave proteoglycans and collagen molecules [53]–[55].

A limitation of the current study is that we can also observe changes in cell density and gene expression patterns even in the SPL group. Ex vivo culture conditions cannot fully mimic normal in vivo conditions. SPL clearly retains the native disc features much better compared to unloading or overloading [29]. Yet, based on changes in gene expression compared to base line, it should be considered that a slow dedifferentiation process occurs also in the SPL condition. This further warrants the importance of reporting both a baseline (day 0) control as a well as a culture control in culture model experiments, as every culture condition will have some effect on the IVDs characteristics regardless of the loading conditions. Furthermore, we did not directly measure proteolytic activity in the IVDs or quantify (breakdown) products of such activity in the culture medium. Therefore, we have to speculate whether observed loss in matrix content was due to the MMPs and ADAMTS5 (as shown to be overexpressed in the PCR data) or if other processes were involved. Hence, further studies on the level of protein production and enzyme activities at more frequent intervals during culture (days rather than weeks) are warranted [56].

To summarize, we found that mechanical overloading evokes degenerative changes at a biomechanical, histological and cellular level in caprine IVDs during the culture period. The course and degree of damage observed in the distinct regions of the disc differ between dynamic and static overloading. Our results support the hypothesis that mechanical overloading can initiate IVD degeneration. Several mediators of catabolic and remodeling activities are triggered by overloading of the IVD (i.e. ADAMTS 5, MMP 13 and 14, IL 1 and 8). These could be potential therapeutic targets for the prevention and/or treatment of (load-induced) DDD [44], [57], [58]. We anticipate that treatments blocking the activity of pro-inflammatory or remodeling enzymes could show efficacy on the delay or attenuation of load-induced disc degeneration [59]–[61]. Whether anti-interleukin or anti-ADAMTS treatment can halt the progression of degeneration over a prolonged period of time, has yet to be elucidated in a large animal in vivo model. Furthermore, the overall net effect of overloading on the biomechanical and matrix level cannot be inferred by such therapy alone. Regeneration of disc matrix and recovery of biomechanical properties should not be expected without restoration of the mechanical environment. Therefore, inclusion of a pro-anabolic, matrix replenishing or substituting agent (cells, (hydro)gels, polymers) in the intervention seems imperative.
